# Clinical Features and Course of Ocular Toxocariasis in Adults

**DOI:** 10.1371/journal.pntd.0002938

**Published:** 2014-06-12

**Authors:** Seong Joon Ahn, Se Joon Woo, Yan Jin, Yoon-Seok Chang, Tae Wan Kim, Jeeyun Ahn, Jang Won Heo, Hyeong Gon Yu, Hum Chung, Kyu Hyung Park, Sung Tae Hong

**Affiliations:** 1 Department of Ophthalmology, Seoul National University Bundang Hospital, Seoul National University College of Medicine, Seongnam, Gyeonggi-do, Korea; 2 Department of Parasitology and Tropical Medicine, Institute of Endemic Diseases, Seoul National University College of Medicine, Seoul, Korea; 3 Department of Internal Medicine, Seoul National University Bundang Hospital, Seoul National University College of Medicine, Seongnam, Gyeonggi-do, Korea; 4 Department of Ophthalmology, Seoul Metropolitan Government Seoul National University Boramae Medical Center, Seoul National University College of Medicine, Seoul, Korea; 5 Department of Ophthalmology, Seoul National University Hospital, Seoul National University College of Medicine, Seoul, Korea; St. George's University, Grenada

## Abstract

**Purpose:**

To investigate the clinical features, clinical course of granuloma, serologic findings, treatment outcome, and probable infection sources in adult patients with ocular toxocariasis (OT).

**Methods:**

In this retrospective cohort study, we examined 101 adult patients diagnosed clinically and serologically with OT. Serial fundus photographs and spectral domain optical coherence tomography images of all the patients were reviewed. A clinic-based case-control study on pet ownership, occupation, and raw meat ingestion history was performed to investigate the possible infection sources.

**Results:**

Among the patients diagnosed clinically and serologically with OT, 69.6% showed elevated immunoglobulin E (IgE) levels. Granuloma in OT involved all retinal layers and several vitreoretinal comorbidities were noted depending on the location of granuloma: posterior pole granuloma was associated with epiretinal membrane and retinal nerve fiber layer defects, whereas peripheral granuloma was associated with vitreous opacity. Intraocular migration of granuloma was observed in 15 of 93 patients (16.1%). Treatment with albendazole (400 mg twice a day for 2 weeks) and corticosteroids (oral prednisolone; 0.5–1 mg/kg/day) resulted in comparable outcomes to patients on corticosteroid monotherapy; however, the 6-month recurrence rate in patients treated with combined therapy (17.4%) was significantly lower than that in patients treated with corticosteroid monotherapy (54.5%, P = 0.045). Ingestion of raw cow liver (80.8%) or meat (71.2%) was significantly more common in OT patients than healthy controls.

**Conclusions:**

Our study discusses the diagnosis, treatment, and prevention strategies for OT. Evaluation of total IgE, in addition to anti-toxocara antibody, can assist in the serologic diagnosis of OT. Combined albendazole and corticosteroid therapy may reduce intraocular inflammation and recurrence. Migrating feature of granuloma is clinically important and may further suggest the diagnosis of OT. Clinicians need to carefully examine comorbid conditions for OT. OT may be associated with ingestion of uncooked meat, especially raw cow liver, in adult patients.

## Author Summary

Toxocariasis is one of America's most common neglected infections of poverty and a helminthiasis of global importance. Little is known about the epidemiologic, demographic, and clinical features of ocular toxocariasis (OT) in adult patients, and the treatment regimen for OT has not been standardized. We conducted a retrospective cohort study examining the clinical features, serologic markers, clinical course of granuloma, probable infection sources, and treatment outcome in 101 adult patients diagnosed clinically and serologically with OT. All the patients had unilateral involvement. Ninety-three (92.1%) and 78 (77.2%) of 101 adult patients had retinal granuloma and intraocular inflammation, respectively. In addition to retinal granuloma, retinal nerve fiber layer defect, epiretinal membrane, vitreous opacity, retinal detachment, macular edema, and macular hole were observed in the eyes with OT. Granuloma in OT can involve all retinal layers, and its intraocular migration was observed in 15 patients (16.1%). Among the 101 patients, 69.6% and 11.6% showed elevated immunoglobulin E levels and eosinophilia, respectively. We believe that OT may be associated with ingestion of uncooked meat, especially cow liver, in adult patients. Furthermore, we suggest that combined albendazole and corticosteroid therapy may reduce intraocular inflammation and recurrence.

## Introduction

Toxocariasis is a globally prevalent illness caused by infestation of the parasite *Toxocara canis* or *Toxocara cati* larvae, which is the most ubiquitous gastrointestinal helminth in dogs and cats [1], [2]. Human beings generally become infected through ingestion of embryonated eggs from contaminated sources such as soil or improperly cooked paratenic hosts [1], [2]. In addition, pet owners can sometimes be accidentally infected by their dogs or cats. After a human ingests the eggs, the eggs hatch in the small intestine and release parasitic larvae. These larvae then penetrate the intestinal wall, enter the circulation, and migrate to organs where they induce inflammatory reactions. Symptoms of the infection vary, depending on the involved organs [1], [2]. Larvae that migrate to the eye cause ocular toxocariasis (OT), which is relatively uncommon and occurs primarily in children. They are most commonly infected through playground and sandbox where contaminated dirt and/or sand may be ingested because of playing habits and poor hygiene [1], [3], [4].

Several OT case series have addressed the demographics, clinical features, and causes of vision loss [3], [5]–[12]. These reports primarily describe young patients, who were under 16 years of age [3], [5]–[11]. However, little is known about the epidemiologic, demographic, and clinical features of OT in adult patients. Most studies had a cross-sectional design, and the clinical course of OT has not been studied extensively. Furthermore, although the mainstay of OT treatment involves steroid use to reduce inflammatory responses [13], the treatment regimen for OT has not been standardized. In particular, the efficacy of combining steroid therapy with anthelmintics has not been determined.

In the present study, we aimed to elucidate the clinical features and course of OT in 101 adult patients with OT, in whom a *Toxocara* infection was confirmed with ELISA serum testing for IgGantibody to the *Toxocara* larva crude antigen [2], [13], [14]. In addition to the ELISA titers, complete blood count (CBC) and serum immunoglobulin E (IgE) levels were obtained in each patient to identify the hematologic/immunologic indicators of OT. Furthermore, to determine the potential sources of *Toxocara* exposure, history of pet ownership, occupation, and raw meat ingestion of the patients were investigated and compared to those of healthy controls. In addition, spectral domain optical coherence tomography (SD-OCT) was performed to investigate OT-related pathologic retinal changes.

## Methods

### Patients and Diagnosis

A retrospective cohort study was conducted in all consecutive adult (>20 years old) patients diagnosed with OT at 3 institutions (Seoul National University Hospital, Seoul National University Bundang Hospital, and Seoul Metropolitan Government Seoul National University Boramae Medical Center) between January 2009 and June 2013.

A clinical diagnosis of OT was made based on (1) typical clinical features of OT [1], [3], [4], (2) positive results by serologic testing, and (3) exclusion of other possible causes of granuloma such as ocular toxoplasmosis (absence of *Toxoplasma*-specific IgG and IgM), sarcoidosis (absence of hilar adenopathy or upper lobe disease on chest radiography, absence of skin lesions suggesting sarcoidosis, absence of hypercalcemia or nephrocalcinosis, and normal levels of angiotensin-converting enzyme), tuberculosis (negative results on interferon gamma release assays, absence of serpiginous choroiditis or retinal vasculitis suggesting ocular tuberculosis, and clinical response to topical/systemic steroid without anti-TB medication), and fungal infection (absence of disseminated fungal diseases, no history of penetrating ocular trauma or surgery within a 6-month period, absence of retinal hemorrhage, which is often observed in eyes with fungal infection but seldom observed in eyes with OT, and clinical response to topical/systemic steroid without anti-fungal agents). The typical clinical features of OT included the presence of a peripheral granuloma (focal, white peripheral nodule with pigmentary scarring or traction retinal detachment), posterior pole granuloma (focal, white nodule with or without posterior pole variable pigmentation), or nematode endophthalmitis (diffuse intraocular inflammation and serology results only positive for *Toxocara*) [1], [3], [4].

### Patient Evaluation

Among the patients with clinical OT, specific IgG antibody titers were measured by indirect ELISA, based on the *T. canis* larva crude antigen [14]. The mean titer of 2 ELISA tests was used in analyses. An ELISA titer of ≥0.250 was considered serologically positive since a previous study to determine the sensitivity and specificity of ELISA testing in patients with toxocariasis showed that a cut-off optical density of 0.250 has a sensitivity and specificity of 92.2% and 86.6%, respectively [14]. The ELISA test was performed on serum in all the patients and on a 1-ml undiluted vitreous sample (obtained during vitreous surgery) in 9 patients who were treated with vitreoretinal surgery. Additionally, a CBC was performed and serum total immunoglobulin-E (IgE) was examined to evaluate any serologic/immunologic abnormalities. The results of abdominal computed tomography (CT) and chest CT were analyzed in this study, if they were performed within 6 months of OT diagnosis. From these images, the prevalence of granulomas or abscesses in other organs such as the lung or liver, as determined by a trained radiologist, were determined.

Best-corrected visual acuity (BCVA), intraocular pressure (IOP), slit-lamp biomicroscopy findings, and dilated fundus examination findings were reviewed in all the patients. Inflammation in the anterior chamber and vitreous chamber was graded based on the number of cells in a 1×1 mm slit beam under maximal light intensity and magnification [15]. Briefly, grade 0 indicated <1 cell; grade 0.5+, 1 to 5 cells; grade 1+, 6 to 15 cells; grade 2+, 16 to 25 cells; grade 3+, 26 to 50 cells; and grade 4+, >50 cells. Funduscopic findings were photographed with a Kowa VX-10 fundus camera (Kowa Co Ltd, Tokyo, Japan). Changes in granuloma size and location were evaluated using photographs from each follow-up visit. SD-OCT (Spectralis, Heidelberg engineering, Heidelberg, Germany) was performed to sectionally image the retina and view pathologic changes in eyes with granuloma and other vitreoretinal complications.

### Treatment of Toxocariasis

Patients with OT were treated with drugs or surgery based on symptom severity, inflammation, and retinal comorbidities. Drug therapy involved corticosteroids when intraocular inflammation was present. Systemic (oral prednisolone; 0.5–1 mg/kg/day loading dose and tapering) and topical (prednisolone acetate 1% four times a day) corticosteroids were used depending on the site of inflammation. Patients with eosinophilia or elevated serum IgE level were treated with albendazole (400 mg twice a day for two weeks). For patients with retinal comorbidities requiring surgery such as visually significant epiretinal membrane, vitreous opacity obscuring visual axis, and tractional or rhegmatogenous retinal detachment, pars plana vitrectomy was performed. Patients were separated into 4 groups, based on the medical treatment they received (i.e., combined steroid and albendazole, albendazole only, steroids only, or no treatment). For patients with a ≥3-month follow-up period, treatment response was evaluated based on clinical characteristics observed at the follow-up visits. These included BCVA, intraocular inflammation grades, symptom improvements, and recurrence rates. Recurrence was defined as returning intraocular inflammation or new granuloma development. Treatment outcomes were assessed in each treatment group.

### Investigation of the Probable Infection Sources

For investigation of the probable infection sources of OT, we conducted a standardized interview and ensured complete responses through improved understanding of the participants to enhance the validity of the interview. During a face-to-face interview, a trained interviewer (medical doctor) used standardized interview procedures to collect data concerning history of eating raw animal tissues and contact with animals and soil during the period between January 2011 and June 2013. Both patients and controls were asked the same set of questions, including puppy/kitten exposure; history of ingestion of raw animal liver, raw meat, and raw animal blood; and occupation-associated contact with animals or soil. The data obtained also included the time of ingestion and the species of animals. For the interview, the questions and uniform nonverbal signals were presented in exactly the same way by one trained interviewer to avoid introducing biases into the responses.

Patients who visited our clinics during January 2011 and June 2013 and showed no abnormal ocular findings on complete ophthalmic examination were selected as controls. Among 59 control subjects who responded to our interview, 50 were matched for age (within 3 years) and sex with the 52 patients who responded to our interview. Between the included controls (n = 50) and the others (n = 9), there were no significant differences in demographic features and probable infection sources, except age (51.0±11.4 in the included controls and 28.3±19.6 in the excluded, P<0.001).

### Statistical Analyses

Descriptive statistical analyses were performed on the demographic data, clinical features, funduscopic and OCT findings, serologic markers, systemic involvement, and granuloma clinical course. Snellen BCVA measurements were converted into logarithmic minimum angle of resolution (logMAR) equivalent values for statistical analysis. The association between funduscopic and OCT findings and granuloma location was assessed by a chi-square test, which compared the frequencies of funduscopic findings in patients with posterior pole granulomas to those with peripheral granulomas. Treatment outcomes were compared between and within groups using the Wilcoxon signed-rank test and Mann–Whitney test, respectively. Continuous and interval data are reported as mean ± standard deviation. Statistical analyses were performed using SPSS for Windows (Ver. 18.0, Statistical Package for the Social Sciences, SPSS Inc., Chicago, IL), and a P value<0.05 was considered statistically significant.

### Ethics Statement

The Institutional Review Board approved this study (Approval #: B-1101/120-102) and all patient data were anonymized for the analysis. The study adhered to the tenets of the Declaration of Helsinki.

## Results

### Demographic and Clinical Characteristics

The demographic and clinical features of the patients are presented in Table 1. Most OT patients were men (76 of 101, 75.2%), and the mean presentation age was 51.7±12.6 years (range: 21–77 years). Three of the 101 patients (3.0%) were known to have been infected with *Toxocara* before they were diagnosed with OT. In the other 98 patients, OT was the first symptom of toxocariasis. Systemic involvement of the *Toxocara* granulomas is also summarized in Table 1. CT images revealed liver (17.6%) or lung (42.9%) granulomas in a significant proportion of our patients.

**Table 1:** Demographics, clinical characteristics, and serologic markers in adult patients with ocular toxocariasis.

	Number of patients (%)
Male:Female	76:25 (75.2:24.8)
Age at presentation, years	51.7±12.6 (range: 21–77)
Previously diagnosed with visceral toxocariasis	3 (3.0%)*
Follow-up period, months	15.0±17.1 (range: 2–61)
**Residence**	
Urban:Rural	76:25 (75.2:24.8)
**Granuloma**	93 (92.1%)
Posterior pole	47 (50.5%)
Peripheral	41 (44.1%)
Macula	16 (17.2%)
Optic nerve	2 (2.2%)
Combined	5 (5.4%)
**Intraocular inflammation**	78 (77.2%)
Anterior uveitis	2 (2.6%)
Intermediate uveitis	53 (67.9%)
Posterior uveitis	13 (16.7%)
Panuveitis	10 (12.8%)
**Vitreoretinal comorbidities**	
Associated retinal nerve fiber layer defect	32 (31.7%)
Epiretinal membrane	27 (26.7%)
Vitreous opacity	22 (21.8%)
Tractional/rhegmatogenous retinal detachment	13 (12.9%)
Pigmentary scarring	8 (7.9%)
Macular edema	4 (4.0%)
Macular hole	2 (2.0%)
**Serologic markers**	
Serum *Toxocara* IgG titer† (ELISA)	0.398±0.115 (range: 0.254–0.737)
Vitreous *Toxocara* IgG titer (ELISA)	0.162±0.162 (range: 0.005–0.431)
Eosinophil count	288±387 (range: 12–2219)
Eosinophilia (>500 cells/µl or ≥10% of total WBC)	10/86 (11.6%)
Immunoglobulin E (IgE)	566±741 (range: 16.6–2650)
Hyper-IgE (serum IgE level >100 unit/ml)	39/56 (69.6%)
**Systemic involvement**	
Liver granuloma in abdominal CT‡	3/17 (17.6%)
Lung granuloma on chest CT‡	6/14 (42.9%)
Lung granuloma on chest radiography‡	4/72 (5.6%)
Brain granuloma‡	1/12 (8.3%)

ELISA = enzyme-linked immunosorbent assay, WBC = white blood cell.

Continuous variables are denoted as mean ± standard deviation.

*Lung granuloma (n = 1), eosinophilia (n = 2).

† An ELISA titer of ≥0.250 was considered serologically positive.

‡ Confirmed by trained radiologists.

Ninety-three of 101 patients (92.1%) were diagnosed with OT based on the presence of a retinal granuloma. Eight patients (7.9%) were diagnosed based on diffuse intraocular inflammation and a positive *Toxocara* antibody (ELISA). Of the 8 patients with nematode endophthalmitis, OT was confirmed in 2 patients, based on the presence of a peripheral granuloma, which became visible only after vitreous opacities had been cleared by vitrectomy. Depending on the location of the granuloma, *Toxocara* granuloma was classified as posterior pole (47 eyes, 50.5%), peripheral (41 eyes, 44.1%), or combined (both posterior pole and peripheral granulomas, 5 eyes, 5.4%). Intraocular inflammation was observed in 78 eyes (77.2%), most of which had intermediate uveitis (53 eyes, 67.9%). All OT cases were unilateral. Mean BCVA changed from 0.51 (20/64 Snellen equivalent) ±0.65 (range: no light perception [NLP] to 30/20) at baseline to 0.45 (20/56 Snellen equivalent) ±0.65 (range: NLP to 30/20) at the final visit. Seventeen (16.8%) and 14 (13.9%) patients had severe vision loss (BCVA<20/200 Snellen equivalent) at baseline and the final visit, respectively. Possible causes of vision loss in patients with OT include retinal damage caused by granuloma itself, comorbidities of OT (Table 1), and intraocular inflammation. Eosinophilia (>500 eosinophils/µl peripheral blood or ≥10% of total white blood cell count [16]) at the time of diagnosis was noted in 10 of 86 patients (11.6%) in whom CBC results were available. Increased serum IgE level (>100 unit/ml) was noted in 39 of 56 patients (69.6%). Mean ELISA titer for serum *Toxocara* IgG was 0.398±0.115 (range: 0.254–0.737).

### Ocular Findings and Clinical Course

**Figure 1:**
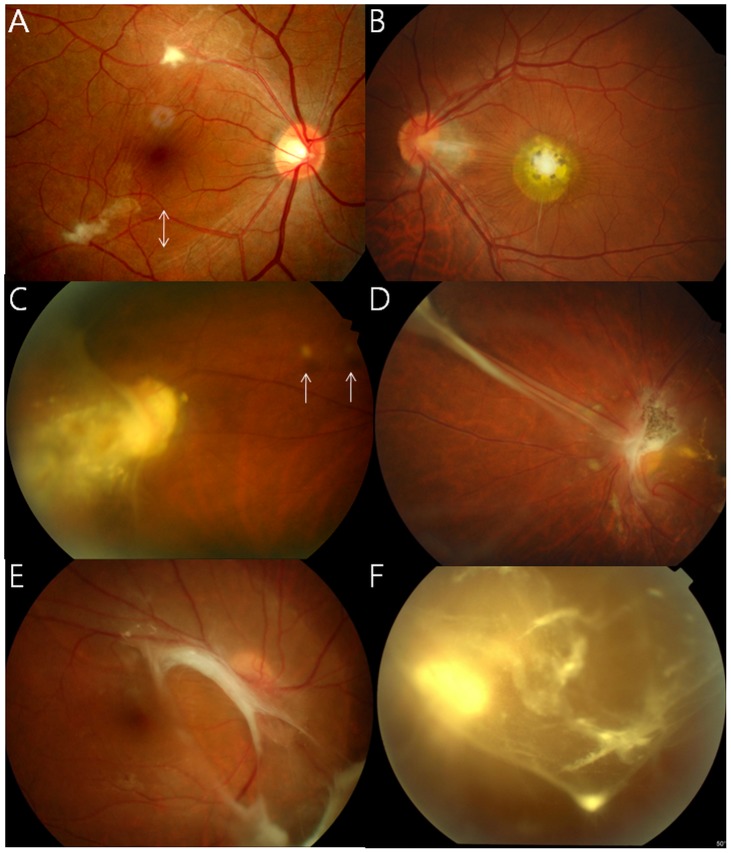
Funduscopic findings of ocular toxocariasis: Small amorphous posterior pole granulomas (A, B) with retinal nerve fiber layer defects (arrows), peripheral granulomas with round vitreous debris (C, arrow) and a tractional retinal fold (D), and vitreous findings, including tractional retinal detachment (E), whitish dot-like vitreous opacities, and vitreous veil (F).

**Figure 2:**
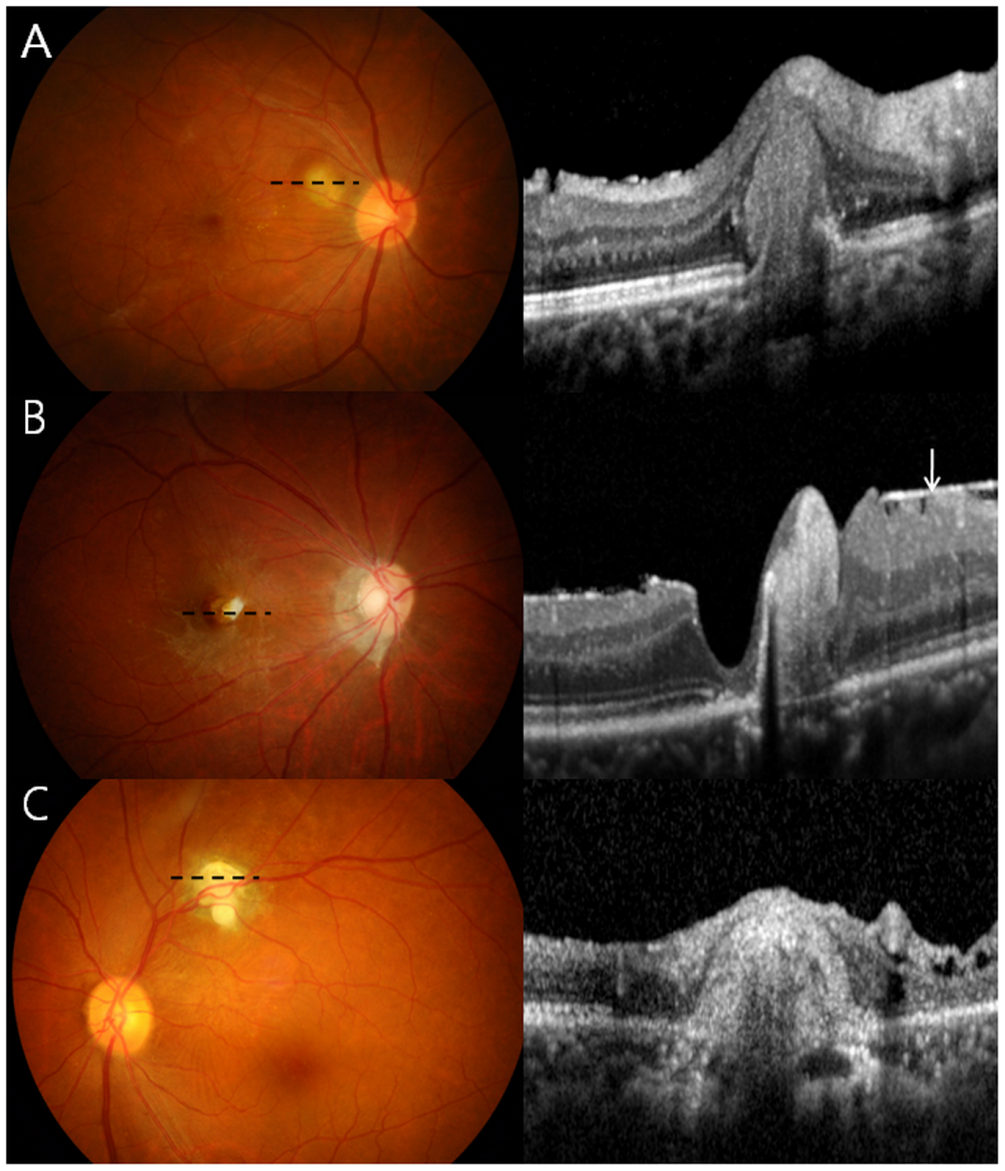
Spectral domain optical coherence tomography (SD-OCT) shows granulomas in the outer (A), inner (B), and all retinal layers (C) as reflective masses with posterior shadowing. The masses remarkably distort the retina and may be accompanied by the epiretinal membrane (B, arrow) and photoreceptor disruption (within two arrowheads). Dotted lines denote the scanned area.

**Figure 3:**
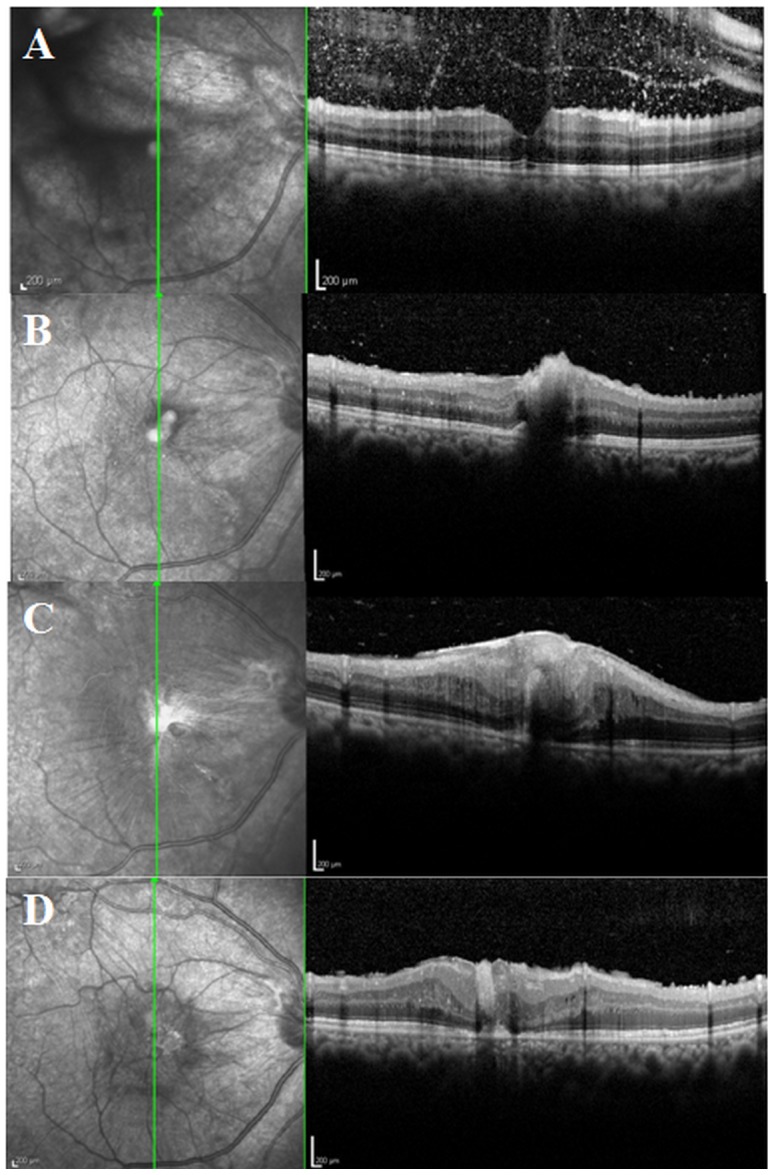
Spectral domain optical coherence tomography (SD-OCT) images showing progressive retinal damage by ocular toxocariasis granulomas. (A) Before discontinuous migration, a normal macular structure is visible, but scattered hyper-reflective dots indicate vitritis. (B) After discontinuous migration from the peripheral retina to the macula, a macular granuloma is observed in the inner retinal layers. (C) One month later, the inner retinal layers show severe distortion, a thick epiretinal membrane is present, and the granuloma extends to the outer retinal layers. (D) Three months after vitrectomy and epiretinal membrane removal, structural damage has not completely resolved.

A few photographs demonstrating retinal and vitreous findings are shown in Figure 1. In addition to retinal granuloma, patients with OT showed retinal nerve fiber layer (RNFL) defect (Figure 1A) in 32 of 101 eyes (31.7%), epiretinal membrane (ERM, Figure 2B and 3C) in 27 eyes (26.7%), vitreous opacity (Figure 1F) in 22 eyes (21.8%), retinal detachment (Figure 1E) in 13 eyes (12.9%), macular edema in 4 eyes (4.0%), and macular hole in 2 eyes (2.0%), as summarized in Table 1. Table 2 shows the association between these vitreoretinal comorbidities and the location of granuloma (posterior pole or peripheral retina). For example, eyes with a posterior pole granuloma had more frequent RNFL defects (53.2% vs. 7.3%, P<0.001) and ERMs (40.4% vs. 14.6%, P = 0.007) than eyes with a peripheral granuloma. In addition, vitreous opacity was observed more often in eyes with a peripheral granuloma than in those with a posterior pole granuloma (31.7% vs. 12.8%, P = 0.031).

**Table 2:** Association between granuloma location and comorbidities in eyes with ocular toxocariasis.

	Posterior pole (n = 47)	Peripheral (n = 41)	*P* †
Associated RNFL defect	25 (53.2%)	3 (7.3%)	<0.001
Epiretinal membrane*	19 (40.4%)	6 (14.6%)	0.007
Vitreous opacity	6 (12.8%)	13 (31.7%)	0.031
Tractional/rhegmatogenous retinal detachment	3 (6.4%)	7 (17.1%)	0.178
Pigmentary scarring	5 (10.6%)	2 (4.9%)	0.442
Macular edema*	1 (2.1%)	2 (4.9%)	0.596
Macular hole*	2 (4.3%)	0	0.497

RNFL = retinal nerve fiber layer.

*confirmed by spectral-domain optical coherence tomography.

† 2×2 chi-square test or Fisher's exact test.

Eyes with both of posterior pole and peripheral granuloma (n = 5) were excluded in analyses.

Pathologic retinal changes were visible on SD-OCT images, which showed a moderately hyper-reflective round mass that sometimes had posterior shadowing (Figure 2). Granulomas were observed in almost all retinal layers. Secondary ERM was also commonly seen, and Figure 3 shows the course of retinal damage that leads to vision loss from granuloma and other OT-associated retinal pathologies.

**Figure 4:**
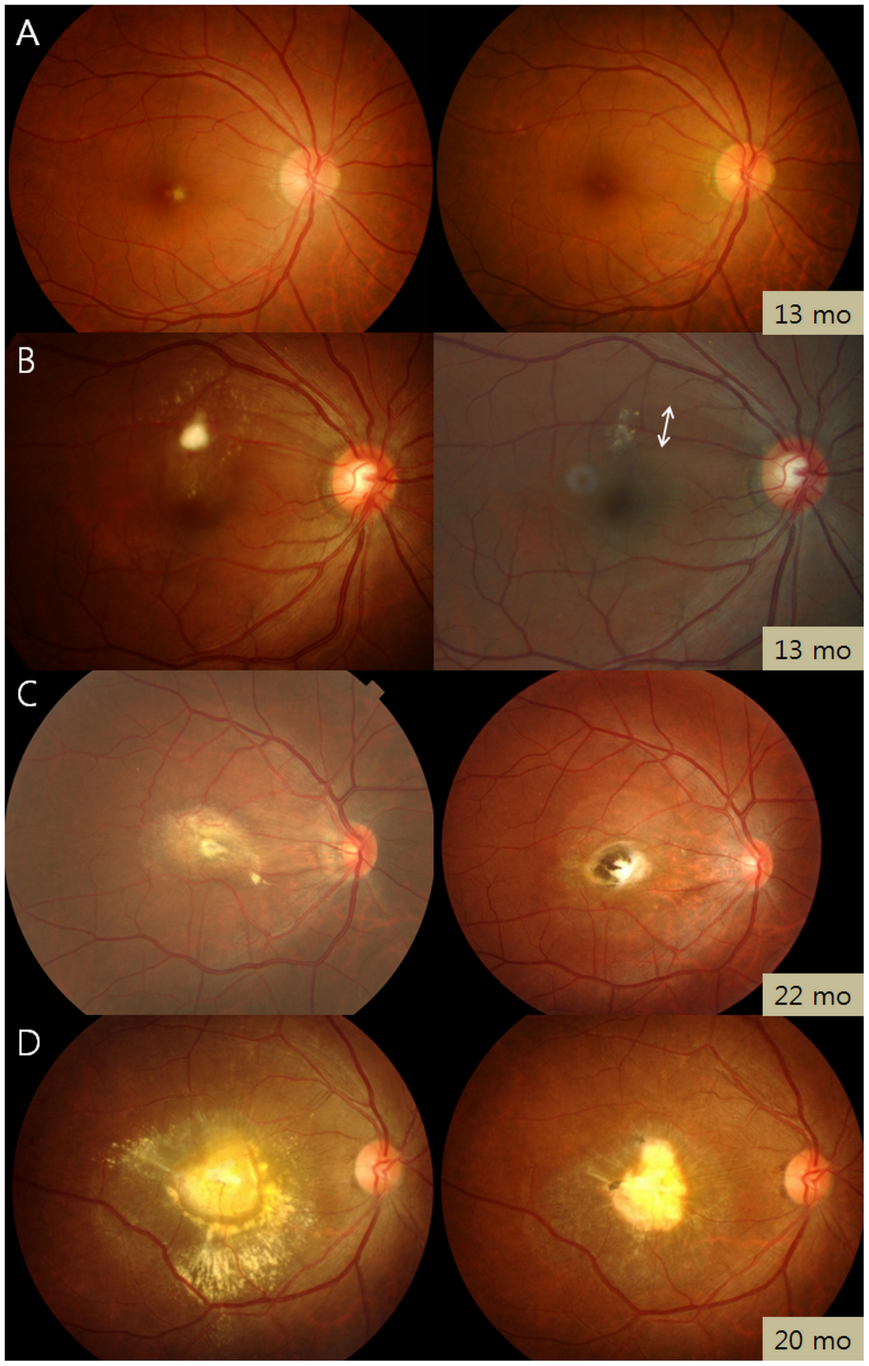
Clinical course of granuloma in ocular toxocariasis. Fundus photographs taken at diagnosis (left column) and the last visit (right column) show several patterns. Complete resolution (A), partial resolution (B), partial resolution of retinal infiltrate with pigmented scar (C), and the remains of an inactive granuloma (D). Each photograph is labeled with the follow-up time in months (mo). Double arrow in B denotes retinal nerve fiber layer defect.

**Figure 5:**
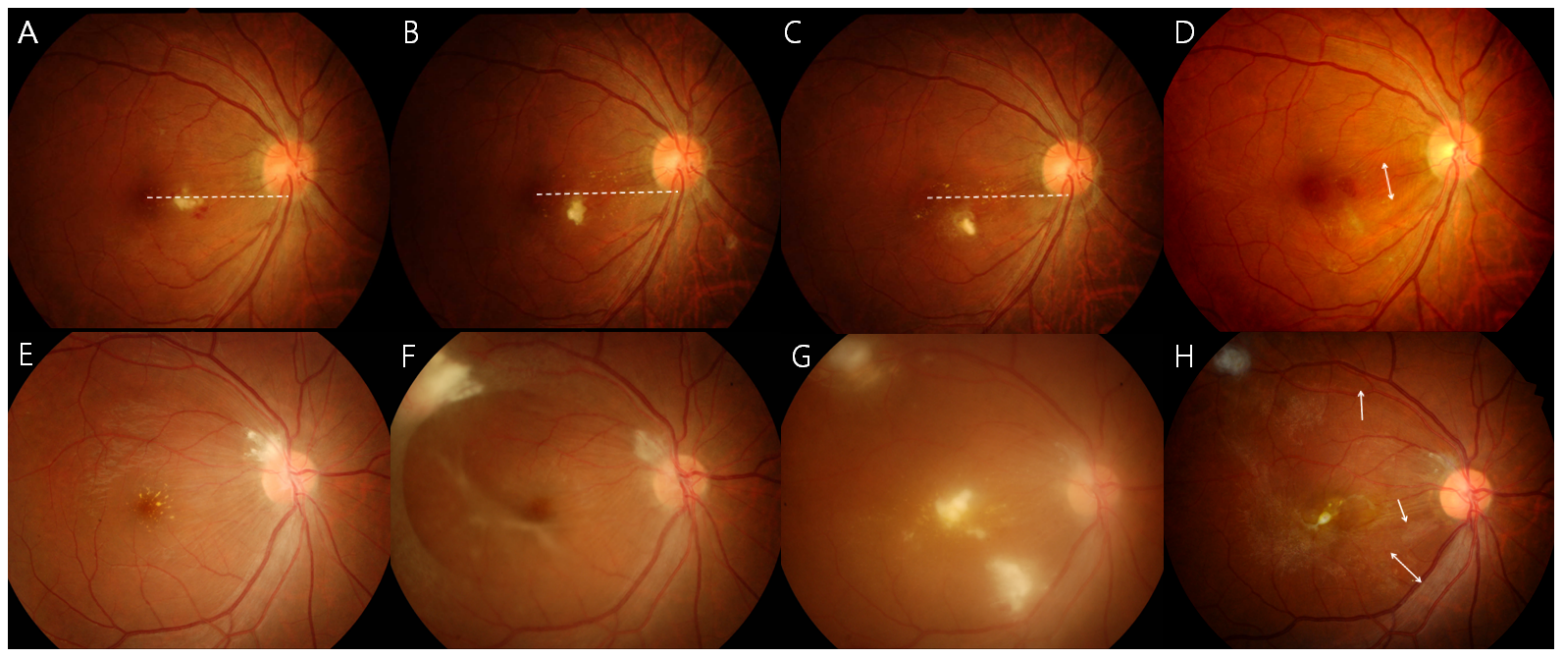
Continuous (A–D) and discontinuous (E–H) migration of *Toxocara* granuloma. (A) The granuloma shape is horizontally long, and the larva lies at the same level as the macula at the time of diagnosis. (B) Gradual inferior granuloma migration is observed. (C) The granuloma has turned toward the inferonasal side. The dotted line denotes a reference line connecting the fovea and inferior optic disc margin. (D) Retinal nerve fiber layer defect (double arrow) remains along the granuloma migration path 41 months after diagnosis. (E) Peripapillary granuloma with macular hard exudate. (F) Four months later, a new granuloma appears along the superotemporal arcade and a tractional membrane extends from the granuloma to the superotemporal vascular arcade and to the inferotemporal retina. A tractional membrane is also noted around the macula. (G) One month later, 2 novel granulomas appear in the macula and inferotemporal retina. (H) Ten months later, the inferior granuloma disappears and the macular granuloma is decreased in size, but multiple retinal nerve fiber layer defects remain (arrows).

Three patterns were noted in the clinical course of *Toxocara* granuloma: complete/partial granuloma resolution, persistent granuloma, and granuloma migration (Table 3). Granulomas completely or partially resolved in 36 of 93 patients (38.7%), with approximately half of these resulting in pigmentary scarring (Figure 4). Forty-two patients (45.2%) showed no significant changes in the size, number, or location of granulomas. Continuous or discontinuous granuloma migration within the eye was observed in 15 patients (16.1%; Figure 5). In continuous migration (12 eyes, 12.9%), the *Toxocara* granuloma migrated but remained adjacent to the originally observed location. However, in discontinuous migration (4 eyes, 4.3%), the granulomas moved discontinuously (relocated far from the originally observed location), increasing the total number of granulomas. One patient showed both types of intraocular migration.

**Table 3:** Clinical course of granuloma in adult patients with ocular toxocariasis.

Variable	Number of Patients (%)
**Complete or partial resolution**	**36 (38.7%)**
Pigmentary scar change	18 (19.4%)
**Persistent granuloma**	**42 (45.2%)**
**Migration of granuloma**	**15 (16.1%)**
Continuous	12 (12.9%)
Discontinuous	4 (4.3%)

### Treatment Outcomes

Table 4 shows treatment responses to the various OT medical therapies. Four types of treatments—combined corticosteroid and albendazole use, corticosteroid use only, albendazole use only, and no treatment—were performed for our patients. The use of corticosteroids significantly decreased the degree of intraocular inflammation (Table 4), but there was no significant improvement in BCVA 3 months after the initiation of drug therapy in any group. In patients with active intraocular inflammation, there was no significant difference in changes in inflammation grade (*P* = 0.619), BCVA (*P* = 0.445), or symptomatic improvement (*P* = 0.274) between patients treated with only corticosteroids and those receiving a combination of corticosteroid and albendazole (Figure S1). In those without active intraocular inflammation, no significant difference was noted in the changes in inflammation grade (P = 1.00), BCVA (P = 0.855), and symptoms (P = 0.206) between patients treated with (Albendazole only group) and without albendazole (No treatment group). However, the 6-month rate of recurrence was significantly lower in the combination treatment group (17.4%) than in the steroid only group (54.5%, *P* = 0.045). In eyes without active inflammation, however, no recurrences were observed in both the Albendazole only and No treatment groups.

**Table 4:** Treatment outcome in patients with ocular toxocariasis who were followed up for ≥3 months.

Treatment modalities	Grade of ocular inflammation* (No. of grade ≥1, %)			Best-corrected visual acuity (logMAR)			Symptomatic improvement for 3 months (%)	Recurrence during 6-month period† (%)
	Before Treatment	Three months after treatment	*P*	Before treatment	Three months after treatment	*P*		
Corticosteroid and albendazole (n = 23)	1.22±0.42 (23, 100%)	0.13±0.34 (3, 13.0%)	<0.001	0.43±0.50	0.33±0.54	0.24	14/23 (60.9)‡	4 (17.4)
Corticosteroid only (n = 11)	1.27±0.47 (11, 100%)	0.27±0.47 (3, 27.3%)	0.002	0.26±0.24	0.24±0.16	0.86	4/11 (36.4)‡	6 (54.5)
Albendazole only (n = 12)	0.083±0.29 (1, 8.3%)	0 (0)	0.32	0.39±0.67	0.44±0.77	0.18	3/9 (33.3)‡	0
No treatment** (n = 11)	0	0	N/A	0.61±0.76	0.64±0.82	1.0	0/8‡	0

N/A = not applicable.

P values were obtained using the Wilcoxon signed-rank test.

* Standardization of uveitis nomenclature (SUN) working group's grading scheme, based on the number of inflammatory cells present in a 1×1 mm slit beam of maximal intensity (grade 0: <1 cell, grade 0.5+: 1–5 cells, grade 1+: 6–15 cells, grade 2+: 16–25 cells, grade 3+: 26–50 cells, grade 4+: >50 cells).

† Recurrence defined as aggravation of ocular inflammation or occurrence of a new granuloma.

‡ Number of patients showing symptomatic improvement/number of symptomatic patients at baseline.

**The data on grade of ocular inflammation at the time of diagnosis and three months after the diagnosis were used.

Thirty-two of 101 patients (31.7%) were surgically treated due to ERM (n = 19), vitreous opacity (n = 9), and/or retinal detachment (n = 2). The surgical outcomes (i.e., BCVA, anatomic success, symptomatic improvement, and recurrence) in each surgical indication are summarized in Table S1. Anatomic success, defined as complete removal of the ERM, vitreous opacity, or retinal reattachment, was achieved in 13 (68.4%), 8 (88.9%), and 2 (50%) patients with ERM, vitreous opacity, and retinal detachment, respectively.

### Probable Sources of Infection in Adults

Table 5 lists the probable sources of infections in adult patients with OT. In demographic features, no significant differences were observed between the patient and control groups in the mean age (51.6±13.0 in the patient group and 51.0±11.4 in the control group, *P* = 0.81) and sex distribution (men∶women = 38∶12 and 39∶13 in the patient and control groups, respectively, *P* = 0.92). Compared to healthy controls, there were no significant differences in the proportion of patients who had ingested raw animal blood, were exposed to puppies or kittens, or had occupation-associated contact with animals and/or soil. However, raw animal meat (71.2% vs. 52%, odds ratio [OR] = 2.28, *P* = 0.047) or cow liver (80.8% vs. 22.0%, OR = 14.9, *P*<0.001) was ingested significantly more often in OT patients than in normal controls.

**Table 5:** Probable sources of infection in adult patients with ocular toxocariasis.

Variable	Patients (n = 52)	Controls (n = 50)	*P*	OR
Puppy/kitten exposure	10 (19.2%)	7 (14%)	0.48	1.46
**Ingestion of animal product**				
Raw cow liver	42 (80.8%)	11 (22%)	<0.001	14.9
Raw meat of animals	37 (71.2%)	26 (52%)	0.047	2.28
Raw animal (deer) blood	5 (9.6%)	2 (4%)	0.44	2.55
**Occupation**				
Frequent contact with animals or animal products	1 (1.9%)	0	1.00	N/A
Frequent contact with soil	1 (1.9%)	0	1.00	N/A

N/A = not applicable; OR = odds ratio.

## Discussion

Despite being the most prevalent human helminth infection in industrialized countries [17], toxocariasis remains relatively unknown to the public [2]. This study describes the pathologic changes caused by OT-associated retinal granulomas using SD-OCT images. The clinical course of granuloma was also examined, and we showed that intraocular granuloma migration is an important and distinguishing clinical feature of OT. In addition, our study showed an association between OT and ingestion of raw cow liver or uncooked meat. This information may also be helpful in diagnosing OT in adult patients.

Systemic and ocular manifestations of toxocariasis have rarely been reported in the same group of patients, and only a few such cases have been described in the literature [18]. In our study, both ocular larva migrans (OLM) and visceral larva migrans (VLM) were assessed in the same group of patients who had undergone ocular examination and liver or chest CT, although it was not proven that granulomas on CT images were caused by *Toxocara* infection. A significant proportion of our patients had liver or lung granulomas on CT images, and further study is needed to better understand the association between VLM and OLM. Given that the vast majority of patients (98 of 101 patients, 97.0%) with toxocariasis were diagnosed first with OT, a thorough ophthalmologic examination is important for the detection of human toxocariasis.

The present study identified several diagnostic serologic markers of *Toxocara* infection. The standard, current test for diagnosing human toxocariasis is detection of serum anti-toxocara IgG using an indirect ELISA based on the *Toxocara* larva antigen [2], [13]. Testing of intraocular fluids for *Toxocara* antibodies has been helpful in diagnosing toxocariasis in a few previous studies [19]–[21] and in some patients of our study. However, the low positive rates (33%) obtained by using the same cut-off value with serum antibodies (0.250) for vitreous antibodies, may not be acceptable for OT detection. Because there is no consensus on the cut-off titers for vitreous antibodies, further research on the diagnostic capabilities of vitreous ELISA is needed. Our study also revealed that serum IgE level is elevated in about 70% of OT patients. Eosinophilia was not as helpful as serum anti-toxocara IgG (ELISA) or total IgE evaluations, although it may indicate the presence of VLM, as shown in previous reports [2], [13].

*Toxocara*-associated RNFL defects, ERMs, and vitreous opacities are common comorbidities of OT. These features were associated with granuloma location, which suggests that careful examination for these associated complications is necessary in patients with retinal granulomas. Vitreous opacities and ERMs were common causes of vision loss in our OT patients, for which surgical treatment was needed. Secondary ERMs in eyes with OT progressed rapidly, resulting in severe retinal distortion and vision deterioration. SD-OCT revealed retinal granulomas and secondary complications, including ERM and tractional membranes, and thus played a critical role in the clinician's decision to perform surgery. Therefore, in patients with OT, SD-OCT may be an important imaging modality for diagnosis and decision making in clinical settings.

The migration of *Toxocara* larvae from the circulatory system into the posterior segment of the eye causes OT. Although migration is a key feature of *Toxocara* larvae, migration within the eye and its clinical significance have not been studied. In this report, we demonstrated a continuous and a discontinuous pattern of intraocular migration. These patterns have been individually reported in different case reports [22], [23], but their incidences have not been determined in longitudinal studies with a large number of patients. Our study showed that 12.9% and 4.3% of patients had continuous and discontinuous migration, respectively. In addition, our findings show clinical implications of the intraocular migration of *Toxocara* larvae. Continuous migration widened RNFL defects and discontinuous migration increased the number of RNFL defects since localized RNFL defects followed granuloma formation. In one case, discontinuous migration resulted in a macular granuloma, which resulted in significant macular destruction and subsequent vision loss, as well as a secondary ERM (Figure 3).

Although the mainstay treatment for OT is the use of corticosteroids to reduce ocular inflammation, this treatment has not yet been standardized. In our study, compared to steroid monotherapy, a combination of albendazole and corticosteroids resulted in a lower rate of recurrence over 6 months. The efficacy of this combination therapy, i.e., no ocular inflammation recurrence and visual acuity improvement, has been shown previously in one case [24]. Although our results showed no significant difference in BCVA before and after treatment, the reduced risk of OT recurrence favors the use of corticosteroids with albendazole in patients with severe OT. Therefore, we recommend using both corticosteroids and albendazole to minimize severe, recurrent inflammation and associated retinal damage.

Our demographic analyses revealed that OT predominantly occurred in men, and disease transmission from pets was less frequent than that reported previously [3]. Male predominance has been previously reported in Japanese [25] (male∶female ratio = 2.5∶1) and Korean [26] (4∶1) populations. In these studies, the mean age was >30 years, which is much higher than that that in studies conducted in Western countries, in which patients were generally <20 years old. Together, these results suggest that East Asian men may have a toxocariasis-related behavior, for example, the ingestion of raw cow liver, which is served in some restaurants in East Asian countries and is believed to be nutritionally beneficial for the middle-aged. Indeed, most reported cases from East Asia involved middle-aged men with a history of ingesting uncooked meat from infected animals [19], [25]–[28]. Contact with a puppy or kitten, which was reported in 82% of patients in a previous OT study [3], has been considered a major infection source, but was only reported in approximately 20% of our OT patients. This indicates that the infection source may differ based on geographic and behavioral differences [2], and clinicians should consider the local cultural context (e.g., food habits) when identifying the probable infection source in adult patients with OT.

Increasing public awareness about toxocariasis is the first step in reducing human exposure to *Toxocara*. Proper handwashing, limiting children's outdoor activity in sandboxes, appropriate disposal of dog and cat feces, and controlling infections in dogs and cats through deworming are the recommended prevention strategies, especially in children [29]. We suggest that OT may be prevented in many adults by avoiding uncooked meat ingestion, especially raw cow liver. Educating the targeted population (middle-aged, East Asian men) as well as clinicians regarding this disease may be effective in preventing the disease [30]. The Japanese government recently banned restaurants from serving raw cow liver. Although intended to prevent infection with a virulent strain of *Escherichia coli*, the action may also reduce the incidence of toxocariasis, especially if applied in East Asia.

Some limitations of our study should be considered. First, although our study included a relatively large cohort of patients compared to previous studies, it is a retrospective study, with intrinsic drawbacks that may introduce bias. The patients presented at >20 years of age, but it does not necessarily mean adult presentation. Additionally, our results on adult infection source may not necessarily extrapolate to the rest of the world as regional food habits vary widely. The serum IgE level was measured only in 55% of the included patients since we started our investigation of immunologic indicators of OT in October 2010. Although the decision regarding whether a patient with OT would be tested for IgE or not was not made by the clinician, selection bias may not be neglected.

Additionally, comparability issues exist in the comparison of treatment outcomes. The method of treatment was determined based on clinical presentation. In OT patients with active intraocular inflammation but without eosinophilia or elevated IgE levels, the current standard treatment for OT, i.e., corticosteroid, was administered. However, in the OT patients with eosinophilia or elevated IgE levels, anthelmintic treatment combined with corticosteroid was preferred due to the possibility of VLM. In this setting, it may not be feasible to compare treatment outcomes between combination therapy and steroid monotherapy groups; similarly, in patients without active inflammation, it may not be feasible to compare albendazole monotherapy and no treatment groups. However, as there were no differences in the baseline ocular characteristics between the two treatment groups, the ocular treatment outcomes could be compared despite the limitation. Further prospective randomized trials are required to compare treatment outcomes between the groups. Despite these limitations, our analyses may be valuable since an optimal treatment for OT has not yet been determined and the role of anthelmintics in OT has not been evaluated.

Another limitation of our study is related to the method of interview used for the investigation of probable infection sources of OT. We tried to avoid introducing biases in the interview procedures between patients and controls by using a standardized interview protocol. However, clinical diagnosis of the participants was not completely blind to the interviewer, possibly leading to bias.

In conclusion, the present study showed that intraocular granuloma migration is an important and distinguishing clinical feature of OT. Among the serologic markers, total IgE, in addition to anti-toxocara IgG antibody (ELISA), may be useful for the diagnosis of OT. In cases showing severe inflammation, combined corticosteroid and albendazole therapy may reduce inflammation and recurrence of OT. The risk of OT in adults may be reduced by avoiding ingestion of uncooked meat, particularly cow liver; however, further studies are required to validate this suggestion.

## Supporting Information

Checklist S1 STROBE Checklist. Page numbers and sections are described within the brackets in bold.
(DOC)

Figure S1 Comparison of outcomes (A: inflammation, B: visual acuity, C: symptom, D: 6-month recurrence) between combined corticosteroid and albendazole therapy and corticosteroid monotherapy in eyes with active inflammation and that between albendazole monotherapy and no treatment. In eyes with active inflammation, both of the combined corticosteroid and albendazole therapy and corticosteroid monotherapy groups show decreased inflammation (A) and improved best-corrected visual acuities (B). These differences in changes in inflammation grade and visual acuity are not significant between the groups. (C) Symptomatic improvement is similar in both groups, but the 6-month recurrence rate is significantly lower in the combination therapy group. In eyes with inactive inflammation, no significant differences in the outcomes between the albendazole monotherapy and no treatment groups are shown.
(TIF)

Table S1 Indications and outcomes for vitrectomy in patients with ocular toxocariasis in those that followed up for ≥3 months.
(DOCX)
