# Supplementary figures and images for: Clinical Features and Course of Ocular Toxocariasis in Adults
Source: PLoS Negl Trop Dis. 2014 Jun 12;8(6):e2938. doi: 10.1371/journal.pntd.0002938 (PMC4055477; doi:10.1371/journal.pntd.0002938)

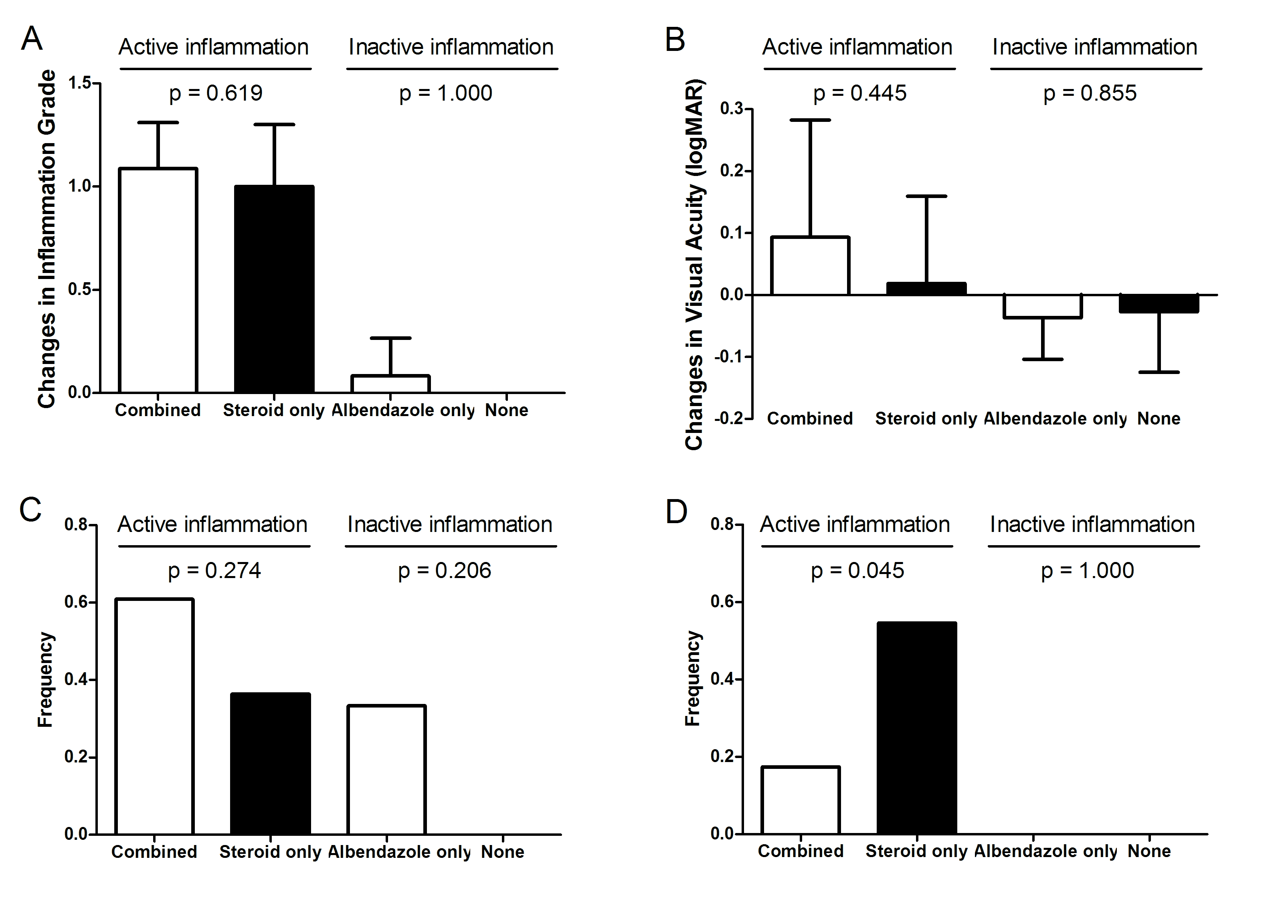

Supplement: Figure S1 — Comparison of outcomes (A: inflammation, B: visual acuity, C: symptom, D: 6-month recurrence) between combined corticosteroid and albendazole therapy and corticosteroid monotherapy in eyes with active inflammation and that between albendazole monotherapy and no treatment. In eyes with active inflammation, both of the combined corticosteroid and albendazole therapy and corticosteroid monotherapy groups show decreased inflammation (A) and improved best-corrected visual acuities (B). These differences in changes in inflammation grade and visual acuity are not significant between the groups. (C) Symptomatic improvement is similar in both groups, but the 6-month recurrence rate is significantly lower in the combination therapy group. In eyes with inactive inflammation, no significant differences in the outcomes between the albendazole monotherapy and no treatment groups are shown. (TIF) [file pntd.0002938.s002.tif]
