# Supplementary material for: Clinical Features and Course of Ocular Toxocariasis in Adults
Source: PLoS Negl Trop Dis. 2014 Jun 12;8(6):e2938. doi: 10.1371/journal.pntd.0002938 (PMC4055477; doi:10.1371/journal.pntd.0002938)
Supplement: Table S1 — Indications and outcomes for vitrectomy in patients with ocular toxocariasis in those that followed up for ≥3 months. (DOCX) [file pntd.0002938.s003.docx]

**Table S1.** Indications and outcomes for vitrectomy in patients with ocular toxocariasis in those that followed up for ≥3 months

| Indications | n (% among surgical cases) | Best-corrected visual acuity (logMAR) | | | Anatomic success*(%) | Symptomatic improvement (%) | Recurrence during 6-month period (%) |
| --- | --- | --- | --- | --- | --- | --- | --- |
|  |  | Before treatment | Three months after surgery | *P* |  |  |  |
| Epiretinal membrane | 19 (59.4) | 0.90 ± 0.84 | 0.52 ± 0.64 | 0.010 | 13 (68.4) | 13/19 (68.4) | 3 (15.8) |
| Vitreous opacity | 9 (28.1) | 0.54 ± 0.55 | 0.25 ± 0.14 | 0.173 | 8 (88.9) | 8/9 (88.9) | 1 (11.1) |
| Tractional/rhegmatogenous retinal detachment | 4 (12.5) | 0.67 ± 0.75 | 0.74 ± 0.83 | 0.715 | 2 (50) | 1/4 (25) | 1 (25) |
| Total | 32 (100) | 0.78 ± 0.76 | 0.48 ± 0.56 | 0.024 | 23 (71.9) | 22 / 32 (68.8) | 5 (15.6) |

For the 3 patients who had >1 surgical indications (e.g. epiretinal membrane and vitreous opacity), the main indication for surgical intervention was selected.

P-values obtained using Wilcoxon signed rank test.

*defined as complete removal of epiretinal membrane, complete clearance of vitreous opacities, or retinal reattachment 3 months after surgery.
